# Supplementary material for: Improving topic modeling performance on social media through semantic relationships within biomedical terminology
Source: PLoS One. 2025 Feb 21;20(2):e0318702. doi: 10.1371/journal.pone.0318702 (PMC11845042; doi:10.1371/journal.pone.0318702)
Supplement: S1 Text — (DOCX) [file pone.0318702.s001.docx]

1. **Phecodes and ICD codes for anxiety/depression**

| Phecode | Description |
| --- | --- |
| 296.2 | Depression |
| 296.22 | Major depressive disorder |
| 300 | Anxiety disorders |
| 300.1 | Anxiety disorder |
| 300.11 | Generalized anxiety disorder |
| 300.12 | Agorophobia, social phobia, and panic disorder |
| 300.13 | Phobia |
| 300.2 | Generalized anxiety & phobic disorders |
| 300.3 | Obsessive-compulsive disorders |
| 300.4 | Dysthymic disorder |
| 300.8 | Acute reaction to stress |
| 300.9 | Posttraumatic stress disorder |

**Table 1. Phecode and corresponding descriptions used to identify anxiety/depression-related symptoms.**

| Code Type | Code | Description |
| --- | --- | --- |
| ICD10CM | F06.4 | Anxiety disorder due to known physiological condition |
| ICD10CM | F32 | Major depressive disorder, single episode |
| ICD10CM | F32.0 | Major depressive disorder, single episode, mild |
| ICD10CM | F32.1 | Major depressive disorder, single episode, moderate |
| ICD10CM | F32.2 | Major depressive disorder, single episode, severe without psychotic features |
| ICD10CM | F32.3 | Major depressive disorder, single episode, severe with psychotic features |
| ICD10CM | F32.4 | Major depressive disorder, single episode, in partial remission |
| ICD10CM | F32.5 | Major depressive disorder, single episode, in full remission |
| ICD10CM | F32.8 | Other depressive episodes |
| ICD10CM | F32.81 | Premenstrual dysphoric disorder |
| ICD10CM | F32.89 | Other specified depressive episodes |
| ICD10CM | F32.9 | Major depressive disorder, single episode, unspecified |
| ICD10CM | F33 | Major depressive disorder, recurrent |
| ICD10CM | F33.0 | Major depressive disorder, recurrent, mild |
| ICD10CM | F33.1 | Major depressive disorder, recurrent, moderate |
| ICD10CM | F33.2 | Major depressive disorder, recurrent severe without psychotic features |
| ICD10CM | F33.3 | Major depressive disorder, recurrent, severe with psychotic symptoms |
| ICD10CM | F33.4 | Major depressive disorder, recurrent, in remission |
| ICD10CM | F33.40 | Major depressive disorder, recurrent, in remission, unspecified |
| ICD10CM | F33.41 | Major depressive disorder, recurrent, in partial remission |
| ICD10CM | F33.42 | Major depressive disorder, recurrent, in full remission |
| ICD10CM | F33.8 | Other recurrent depressive disorders |
| ICD10CM | F33.9 | Major depressive disorder, recurrent, unspecified |
| ICD10CM | F34.1 | Dysthymic disorder |
| ICD10CM | F40 | Phobic anxiety disorders |
| ICD10CM | F40.0 | Agoraphobia |
| ICD10CM | F40.00 | Agoraphobia, unspecified |
| ICD10CM | F40.01 | Agoraphobia with panic disorder |
| ICD10CM | F40.02 | Agoraphobia without panic disorder |
| ICD10CM | F40.1 | Social phobias |
| ICD10CM | F40.10 | Social phobia, unspecified |
| ICD10CM | F40.11 | Social phobia, generalized |
| ICD10CM | F40.2 | Specific (isolated) phobias |
| ICD10CM | F40.21 | Animal type phobia |
| ICD10CM | F40.210 | Arachnophobia |
| ICD10CM | F40.218 | Other animal type phobia |
| ICD10CM | F40.22 | Natural environment type phobia |
| ICD10CM | F40.220 | Fear of thunderstorms |
| ICD10CM | F40.228 | Other natural environment type phobia |
| ICD10CM | F40.23 | Blood, injection, injury type phobia |
| ICD10CM | F40.230 | Fear of blood |
| ICD10CM | F40.231 | Fear of injections and transfusions |
| ICD10CM | F40.232 | Fear of other medical care |
| ICD10CM | F40.233 | Fear of injury |
| ICD10CM | F40.24 | Situational type phobia |
| ICD10CM | F40.240 | Claustrophobia |
| ICD10CM | F40.241 | Acrophobia |
| ICD10CM | F40.242 | Fear of bridges |
| ICD10CM | F40.243 | Fear of flying |
| ICD10CM | F40.248 | Other situational type phobia |
| ICD10CM | F40.29 | Other specified phobia |
| ICD10CM | F40.290 | Androphobia |
| ICD10CM | F40.291 | Gynephobia |
| ICD10CM | F40.298 | Other specified phobia |
| ICD10CM | F40.8 | Other phobic anxiety disorders |
| ICD10CM | F40.9 | Phobic anxiety disorder, unspecified |
| ICD10CM | F41.0 | Panic disorder [episodic paroxysmal anxiety] |
| ICD10CM | F41.1 | Generalized anxiety disorder |
| ICD10CM | F41.3 | Other mixed anxiety disorders |
| ICD10CM | F41.8 | Other specified anxiety disorders |
| ICD10CM | F41.9 | Anxiety disorder, unspecified |
| ICD10CM | F42 | Obsessive-compulsive disorder |
| ICD10CM | F42.2 | Mixed obsessional thoughts and acts |
| ICD10CM | F42.3 | Hoarding disorder |
| ICD10CM | F42.4 | Excoriation (skin-picking) disorder |
| ICD10CM | F42.8 | Other obsessive-compulsive disorder |
| ICD10CM | F42.9 | Obsessive-compulsive disorder, unspecified |
| ICD10CM | F43.0 | Acute stress reaction |
| ICD10CM | F43.1 | Post-traumatic stress disorder (PTSD) |
| ICD10CM | F43.10 | Post-traumatic stress disorder, unspecified |
| ICD10CM | F43.11 | Post-traumatic stress disorder, acute |
| ICD10CM | F43.12 | Post-traumatic stress disorder, chronic |
| ICD10CM | F48.8 | Other specified nonpsychotic mental disorders |
| ICD10CM | F48.9 | Nonpsychotic mental disorder, unspecified |
| ICD10CM | F99 | Mental disorder, not otherwise specified |
| ICD10CM | R45.2 | Unhappiness |
| ICD10CM | R45.5 | Hostility |
| ICD10CM | R45.6 | Violent behavior |
| ICD10CM | R45.7 | State of emotional shock and stress, unspecified |
| ICD10CM | Z86.51 | Personal history of combat and operational stress reaction |
| ICD9CM | 293.84 | Anxiety disorder in conditions classified elsewhere |
| ICD9CM | 296.2 | Major depressive disorder, single episode |
| ICD9CM | 296.20 | Major depressive affective disorder, single episode, unspecified |
| ICD9CM | 296.21 | Major depressive affective disorder, single episode, mild |
| ICD9CM | 296.22 | Major depressive affective disorder, single episode, moderate |
| ICD9CM | 296.23 | Major depressive affective disorder, single episode, severe, without mention of psychotic behavior |
| ICD9CM | 296.24 | Major depressive affective disorder, single episode, severe, specified as with psychotic behavior |
| ICD9CM | 296.25 | Major depressive affective disorder, single episode, in partial or unspecified remission |
| ICD9CM | 296.26 | Major depressive affective disorder, single episode, in full remission |
| ICD9CM | 296.3 | Major depressive disorder, recurrent episode |
| ICD9CM | 296.30 | Major depressive affective disorder, recurrent episode, unspecified |
| ICD9CM | 296.31 | Major depressive affective disorder, recurrent episode, mild |
| ICD9CM | 296.32 | Major depressive affective disorder, recurrent episode, moderate |
| ICD9CM | 296.33 | Major depressive affective disorder, recurrent episode, severe, without mention of psychotic behavior |
| ICD9CM | 296.34 | Major depressive affective disorder, recurrent episode, severe, specified as with psychotic behavior |
| ICD9CM | 296.35 | Major depressive affective disorder, recurrent episode, in partial or unspecified remission |
| ICD9CM | 296.36 | Major depressive affective disorder, recurrent episode, in full remission |
| ICD9CM | 300 | Anxiety, dissociative and somatoform disorders |
| ICD9CM | 300.0 | Anxiety states |
| ICD9CM | 300.00 | Anxiety state, unspecified |
| ICD9CM | 300.01 | Panic disorder without agoraphobia |
| ICD9CM | 300.02 | Generalized anxiety disorder |
| ICD9CM | 300.09 | Other anxiety states |
| ICD9CM | 300.10 | Hysteria, unspecified |
| ICD9CM | 300.2 | Phobic disorders |
| ICD9CM | 300.20 | Phobia, unspecified |
| ICD9CM | 300.21 | Agoraphobia with panic disorder |
| ICD9CM | 300.22 | Agoraphobia without mention of panic attacks |
| ICD9CM | 300.23 | Social phobia |
| ICD9CM | 300.29 | Other isolated or specific phobias |
| ICD9CM | 300.3 | Obsessive-compulsive disorders |
| ICD9CM | 300.4 | Dysthymic disorder |
| ICD9CM | 300.5 | Neurasthenia |
| ICD9CM | 300.89 | Other somatoform disorders |
| ICD9CM | 300.9 | Unspecified nonpsychotic mental disorder |
| ICD9CM | 308 | Acute reaction to stress |
| ICD9CM | 308.0 | Predominant disturbance of emotions |
| ICD9CM | 308.1 | Predominant disturbance of consciousness |
| ICD9CM | 308.2 | Predominant psychomotor disturbance |
| ICD9CM | 308.3 | Other acute reactions to stress |
| ICD9CM | 308.4 | Mixed disorders as reaction to stress |
| ICD9CM | 308.9 | Unspecified acute reaction to stress |
| ICD9CM | 309.81 | Posttraumatic stress disorder |
| ICD9CM | 311 | Depressive disorder, not elsewhere classified |
| ICD9CM | 313.0 | Overanxious disorder specific to childhood and adolescence |
| ICD9CM | 313.1 | Misery and unhappiness disorder specific to childhood and adolescence |
| ICD9CM | 313.21 | Shyness disorder of childhood |
| ICD9CM | 313.22 | Introverted disorder of childhood |
| ICD9CM | 313.3 | Relationship problems specific to childhood and adolescence |
| ICD9CM | 313.82 | Identity disorder of childhood or adolescence |
| ICD9CM | 313.83 | Academic underachievement disorder of childhood or adolescence |
| ICD9CM | V11.4 | Personal history of combat and operational stress reaction |

**Table 2. ICD codes and corresponding descriptions mapped from relevant Phecodes to identify anxiety/depression-related symptoms.**
