# Supplementary material for: Improving topic modeling performance on social media through semantic relationships within biomedical terminology
Source: PLoS One. 2025 Feb 21;20(2):e0318702. doi: 10.1371/journal.pone.0318702 (PMC11845042; doi:10.1371/journal.pone.0318702)
Supplement: S2 Text — (DOCX) [file pone.0318702.s002.docx]

**Appendix**

1. **Data Resource**

We retrieved data from Reddit, a prominent and diverse social media platform where people interact and share information on the web [1]. Reddit’s data are structured into topic threads, with each thread featuring a primary post known as a “submission”. These submissions typically consist of a title, body text, and subsequent comments. Topic threads can also be further grouped into user-generated forums, known as ‘subreddits’, where individuals from various backgrounds and interests come together to engage in discussions, share content, and build communities around common topics [2]. In subreddits, users can leave comments and either upvote or downvote any submission or comment. In this study, we extracted the submissions and comments from *r/Cholesterol*, a subreddit created in October 2009, which is the largest community on Reddit for patients with lipid issues, especially hyperlipidemia. At the time of paper writing, *r/Cholesterol* had approximately 13,200 active users, with a broad range of topics (e.g., lab results, dietary and life changes, symptoms, and treatments).

1. **Data preprocessing**

To prepare the collected data for UMLS concept recognition, we combined the content of both the title and body text into a unified text paragraph for each submission. To ensure the accurate recognition of UMLS concepts and to eliminate unnecessary tokens from the text corpus, such as stopwords (e.g., ‘a’, ‘to’, ‘the’), numbers, and punctuation, we retained only nouns, verbs, and adjectives from the corpus. We observed that some single letters (e.g., ‘m’, ‘e’) and special characters (e.g., ‘\#x200b’) in the text corpus were erroneously recognized as nouns, so we manually removed these letters and characters. Following vocabulary cleaning, we proceeded to convert all words to lowercase. These data preprocessing steps were executed using the Python package spaCy (version 3.7.5) [3].

1. **Blind expert review**

We conducted a blind qualitative review from three experts with strong medical and pharmaceutical backgrounds to evaluate the two groups of topics given by **Figure 4(a)** and **Figure 4(b)**. The experts were not aware which group of topics were before or after concept decomposition. The experts were asked to compare the two groups of topics in terms of their quality and clinical interpretability and were also asked to summarize each topic using a few words. Their reviews are summarized as follows:

1. **Expert 1**: Highlights that while both groups in the figures have unexpected elements, the second group (Figure 4(b)) offers greater clinical interpretability. The first group (Figure 4(a)) appears disorganized, with topics encompassing unrelated symptoms and side effects. The second group, however, neatly categorizes topics into clear categories: "diabetes and digestive issues," "anxiety," and "myopathy, depression, and fatigue."
2. **Expert 2**: Notes no significant differences between the two groups, describing the first group as covering "metabolic syndrome disorders" and related complications, affecting various organs. The second group is defined by topics on "comorbidities of metabolic syndrome," "non-muscle related side effects," and a mix of "muscle pain, complications, and non-specific syndromes."
3. **Expert 3**: Finds the topics in the second group more interpretable, as they more clearly delineate distinct adverse effects of statin use. The first group's topics are a mix of "muscle pain, anxiety, gastrointestinal (GI) symptoms, diabetes, confusion, and fatigue" and effects on "the liver and GI symptoms." The second group is organized into "GI symptoms, diabetes, and confusion," "psychiatric adverse effects," and a combination of "muscle pain, depression, and fatigue."
4.
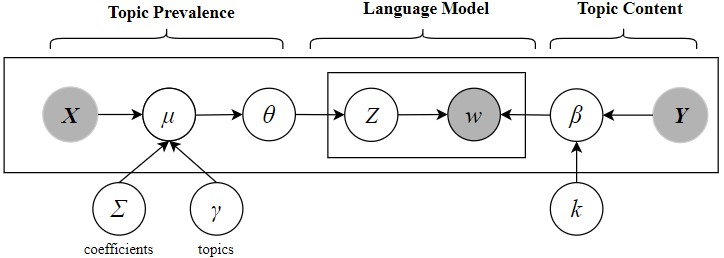
**STM framework and parameters**

**Figure 7. Plate diagram of STM**.

**Figure 7** presents the plate diagram illustrating STM [4]. STM focuses on understanding the thematic structure of a corpus by modeling the relationships between topics and documents, as well as the connections between words and topics.[4] STM characterizes topics as compositions of words, with each word being associated with a topic with a certain probability. Similarly, documents are represented as mixtures of topics, each with its probabilities [5]. STM engages in the estimation of key elements, including topic prevalence (*μ*), the topic-document distribution (*θ*), and the word-topic distribution (*β*). This approach incorporates prior word-topic assignments (*z*) and observed words (*w*) in an iterative process that refines the assignments of words to topics, providing a dynamic and evolving understanding of the intricate relationships between topics, words, and documents within the corpus. The number of topics (*k*) is one of the most important parameters in STM to be determined by researchers. The value of *k* is selected to achieve a substantive interpretation of the outcomes rather than maximization of the fit [6].

**References**

1. Reddit. Wikipedia. 2023. Available: https://en.wikipedia.org/w/index.php?title=Reddit&oldid=1177572582

2. Studying Reddit: A Systematic Overview of Disciplines, Approaches, Methods, and Ethics - Nicholas Proferes, Naiyan Jones, Sarah Gilbert, Casey Fiesler, Michael Zimmer, 2021. [cited 8 Oct 2023]. Available: https://journals.sagepub.com/doi/full/10.1177/20563051211019004

3. Vasiliev Y. Natural Language Processing with Python and spaCy: A Practical Introduction. No Starch Press; 2020.

4. Roberts ME, Stewart BM, Airoldi EM. A Model of Text for Experimentation in the Social Sciences. Journal of the American Statistical Association. 2016;111: 988–1003. doi:10.1080/01621459.2016.1141684

5. Roberts ME, Stewart BM, Tingley D. stm: An R Package for Structural Topic Models. Journal of Statistical Software. 2019;91: 1–40. doi:10.18637/jss.v091.i02

6. Chang J, Gerrish S, Wang C, Boyd-graber J, Blei D. Reading Tea Leaves: How Humans Interpret Topic Models. Advances in Neural Information Processing Systems. Curran Associates, Inc.; 2009. Available: https://proceedings.neurips.cc/paper_files/paper/2009/hash/f92586a25bb3145facd64ab20fd554ff-Abstract.html
