# Supplementary material for: Improving topic modeling performance on social media through semantic relationships within biomedical terminology
Source: PLoS One. 2025 Feb 21;20(2):e0318702. doi: 10.1371/journal.pone.0318702 (PMC11845042; doi:10.1371/journal.pone.0318702)
Supplement: S3 Text — (DOCX) [file pone.0318702.s003.docx]

**I**．**Example submissions for each topic in the final STM:**

Topic 1(metabolic syndrome):

#1: “High cholesterol low hdl？Hello got my test results and my doctor put me on Lipitor 10mg daily. I'm 31 years old high blood pressure taking medication for it now and obese. I'm working on lowering my weight and only drinking water and exercise.

My total cholesterol is 204

HDL cholesterol is 34

LDL cholesterol is 136

triglycerides is 188”

#2: “Statin or Ezetimibe therapy for preventative and overall longevity purposes? Hi r/Cholesterol, will try to keep this short and to the point. I am a 32-year-old male, 5'10"", 150 lbs, whose family has a moderate history of high cholesterol, heart disease, and heart attack. With this in mind, I am hyper-focused on increasing my chances of not going down this same path. [Here is my lipid panel history](https://imgur.com/gallery/M3MYEH9). Not included is my Lipoprotein (a) aka Lp(a) which is 99 nmol/L. I believe my elevated levels are genetic as I have good exercise and diet practices. Back to the point of my post for heart disease prevention and overall longevity in mind, my Cardiologist wants to begin a mild 20mg Statin therapy. I have also read that an Ezetimibe like Zetia is also a good option. Would a combination of both be an option? Ideally, I would take a PCSK9 Inhibitor in addition to a Statin but unfortunately, insurance won't cover PCSK9 and it would cost tens of thousands of dollars.”

#3: “Statin alternatives to discuss with my Dr. Me: 37F, mildly overweight (but not obese), exercise regularly (mix of strength, running, yoga), good diet, high LDL (190; probably genetics although haven not tested yet). My doctor started me on 10mg atorvastatin (Lipitor) every other day. I am a couple weeks in and have been experiencing non-stop reflux/heartburn which is exacerbated by exercise and laying down. Can I continue with this for the rest of my life. I have a message out to my doctor to discuss alternatives, but wanting to know if anyone else with high LDL has found an effective alternative to statins. I would like to make a list to look up info on, formulate questions, and then discuss with my doctor. LDR anyone find an effective alternative to statins for lowering LDL?”

Topic 2 (anxiety and depression):

#1: “Statins affect my mood in a very negative way My doc took me off fenofibrate and put me on rosuvastatin. I've had high triglycerides for a couple of years but my cholesterol had always been good. When I started on rosuvastatin it was tolerable the first day. The second day I was a bit sparky for want of a better word. Very quick to answer people back and not necessarily in a good/appropriate/nice way. The next day it was worse and again the next. I was feeling irrational anger and extreme depression at that point. Everything was pissing me off, making me angry and I did not recognize my own personality. It was also making me extremely hyper, bouncing off the walls. I stopped taking them for a week and my mood slowly returned to normal. I gave it a few more days to make sure, then started taking it again. The same thing is happening again. This time, I also have massive amounts of calf pain in my legs and quite a lot of pain all over too. I have fibromyalgia so have a lot of pain to begin with. This is making it much worse. I'm less concerned about the pain than the mood swing. I can live with pain but my wife and kids cannot live with the personality change. I do not see much on here about a change in mood/behavior when taking statins. Has anyone else had a similar experience and how did you deal with it?”

#2: “Statins and Depression/Mental Health I'm someone with FH (LDL \~260 without any meds) who doesn't respond well to statins. Doesn't respond well in my case means even a 5mg dose of rosuvastatin or 1mg of pitavastatin (livalo) leaves me feeling very mentally unwell within 24hrs of the dose. For me that's anxiety, horrible feeling of hopelessness and very depressed. My Dr is unconvinced it's statins however after many re-challenge i get the same feelings very quickly after taking a statin and trying my best to stay on them for as long as i can. This will go away within about a week of stopping. It's very frustrating as a fit and otherwise healthy guy. As someone with FH i realise the probable need to take a high intensity statin however at this point there's just no way it seems, so it's ezetimibe only. Anybody else had any similar issues? I can see in the literature there are studies that show this kind of outcome, so it does seem to be an issue, if somewhat rare/underreported. Thanks.”

#3: “started Crestor knocked down my cholesterol but causing other issues. Albumin I have had high cholesterol for decades but have been unable to get on statins due to experiencing side effects with all of them. My cholesterol is so high that my doctor is very worried so I started Crestor again for the third time trying in my life, but only taking 2.5 instead of the 5 mg. It knocked my cholesterol down 30 points in 3 weeks and lowered my bad cholesterol as well. However I am experiencing depression, joint pain, raised sugar and sleep disturbance, all of which I have experienced before on statins. and my recent blood test also revealed high a/g and albutum, something new for me. I am very disturbed by this and I just don't know what to do at this point. Insurance won't pay for the newer statins because my doctor and I have fought them for months. I'm just at a loss and at high risk and it's all very concerning”.

Topic 3 (muscle pain):

#1: “I had tried Lipitor in the past but stopped because it caused bad muscle aches. Took my first dose of Crestor (10mg) yesterday and could definitely feel some aches in my joints throughout the day. This morning, I woke up and my entire body is aching. I wanted to ask if anyone else has experienced these symptoms after only a single dose.”

#2: “Has anyone on the Mediterranean diet NO MEDS seen cholesterol improvement? My doctor suggested the Mediterranean diet to try since I didn't handle statins very well (severe muscle pains) and the next non statin medication isn't as beneficial. Soooo..I'm wondering if anyone has tried this approach before?”

#3: “Statin - calf pain? Help with your experience. I've had sudden calf pain that feels vascular in nature but no blood clots after going to emergency ultrasound. I don't think I pulled a muscle. The pain is excruciating in one calf only, now radiating to back of thigh. Been on 5mg of Atorvastin for like a year, about two months ago bumped it to 10mg that was prescribed. I am in so much pain I can't walk. Prior to this, I was in gym 5 days a week, still have a dad bod but I was moving. Could this pain be statin related even if it is just one calf? Edit:. After a misdiagnosed ultrasound and a doctor who gave wrong advice, a stenographer found I had blood clots in a second ultrasound.”
