# Supplementary material for: Improving topic modeling performance on social media through semantic relationships within biomedical terminology
Source: PLoS One. 2025 Feb 21;20(2):e0318702. doi: 10.1371/journal.pone.0318702 (PMC11845042; doi:10.1371/journal.pone.0318702)
Supplement: Attachment — Submitted filename: Response_to_ Reviewers.docx [file pone.0318702.s004.docx]

**Response to Reviewers**

Thank you for the opportunity to respond to comments received for our application, “Improving Topic Modeling Performance on Social Media Through Semantic Relationships within Biomedical Terminology”. We appreciate the committee’s suggestions and positive comments including: “the innovation lies in the combination of UMLS concept recognition, SNOMED CT relationship-based concept decomposition, and traditional topic modeling to create a more robust method for analyzing health-related social media content”; “the paper presents a method for improving the extraction of meaningful health-related themes from social media, which could lead to better patient insights and enhanced clinical decision-making”; “the manuscript is well-structured and clearly written, making the methodology and findings accessible”; etc.

Below, we address the reviewers’ concerns (the line numbers mentioned in this letter are from Revised Manuscript with Track Changes):

**Reviewer 1:**

***Q1: While the introduction does mention some related work (e.g., lines 36-52 discuss various applications of topic modeling in healthcare), the authors don't explicitly compare their approach to these existing methods in terms of performance or outcomes.
Some potential comparisons could include:
a) Traditional topic modeling approaches (e.g., Latent Dirichlet Allocation) on the same dataset without semantic enrichment.
b) Other semantic-enhanced topic modeling methods that have been applied to healthcare social media data.
c) Alternative approaches to extracting health-related information from social media, such as supervised machine learning methods or rule-based systems.***

**Answer**: Thanks for the comments. We have expanded the Introduction to incorporate greater rationale for the choice of STM in introduction section (lines 60-65): “The structural topic model (STM) is one of the most common types of topic modeling approaches used widely in analysis of healthcare data and social media health discussions, as it excels in uncovering latent themes and temporal trends. Unlike other topic modeling approaches, such as Latent Dirichlet Allocation, STM is uniquely able to incorporate document-level metadata, such as user attributes, simultaneously with topic inference, making it particularly advantageous for analyzing social media data in health-related NLP tasks.” In our study, the document-level metadata includes submission creation time, comment count, score for each submission. This integration enhances the interpretability and relevance of the extracted topics. For instance, the STM allows researchers to estimate topic models that include document-level metadata, providing rich ways to explore topics, estimate uncertainty, and visualize quantities of interest [1,2]. In addition, although LDA was popular in many text mining studies, it has been well documented in the empirical literature that LDA is sub-optimal for short texts published in social media and needs to be improved to analyze noisy social media texts [3]. Therefore, for (a): we think the STM applied in our study should perform better than the traditional LDA in our dataset. In addition, our focus is to develop a new semantic-type-based topic modeling approach to cluster more meaningful topics in clinical perspective. Hence, the application of other traditional approaches (e.g., LDA) is not necessary and/or may provide less productive results for this dataset. For (b), our semantic-type-based topic modeling approach generates topics comprised of standard medical concepts, making them more interpretable for clinicians and doctors. This is one of our study’s merits. To date, there does not exist any other semantic-based topic modeling approach that can generate topics comprised of standard medical concepts, whether it has been applied to healthcare social media data or not. For (c), supervised approaches, such as Support Vector Machines (SVMs) and neural networks, are trained on labeled datasets to classify text into predefined topics. Rule-based systems utilize predefined linguistic rules and keyword matching to identify topics within the text. However, these methods have certain limitations compared to unsupervised topic modeling approaches. Supervised methods require extensive labeled data, which can be labor-intensive to produce and may not capture the full range of topics present in dynamic social media content. Rule-based systems, while straightforward, often lack the flexibility to adapt to the evolving language and slang typical of social media platforms. In contrast, topic modeling approaches is more adaptable to the nuances of social media language [3].

***Q2:***

***Introduction:
- Line 36: "Topic modeling is an unsupervised machine learning technique in natural language processing (NLP) used" - Consider rephrasing to improve flow, e.g., "Topic modeling, an unsupervised machine learning technique in natural language processing (NLP), is used"***

**Answer**: Thanks for the suggestion. We have revised this sentence accordingly.

***Q3:***

***Methods:***

***Line 84: "We conducted web scraping of a repository containing dump files to collect the submissions and comments" - Consider providing more details on the ethical considerations of data collection from social media***

**Answer**: Thanks for the suggestion. The ethical considerations of using social media data for healthcare research was added in the Methods section (lines 117-120): “In terms of ethical considerations of using social media data for healthcare research, we ensured adherence to ethical standards and for data collection from social media platforms. Specifically, the extracted social media data was de-identified and no personal characteristics from users were collected.”

***Q4:***

***Results:***

***Figure 2: Consider adding a brief explanation of how to interpret the topic proportions in the figure caption***

**Answer**: Thanks for the suggestion. The brief explanation of the topic proportions has been add to lines 213-214: “Topic proportion is the percentage of the documents associated with a specific topic, indicating its prevalence”.

***Q5:***

***Discussion:***

***Line 315: "Along with these widely recognized topics associated with statin use, we also noted novel findings relative to topic 2:" - Consider elaborating on the potential implications of these novel findings for healthcare providers and researchers***

**Answer**: Thanks for the suggestion. The potential implications of these novel findings has been added to lines 378-381: “The finding highlights the need for healthcare providers to actively monitor and address mental health symptoms, such as anxiety and depression, in patients using statins. They may consider integrating routine mental health screenings into follow-up visits for statin users and providing resources or referrals for mental health support when necessary.”.

***Q6:
Limitations and future work:
- Consider expanding on potential solutions or approaches to address the limitations mentioned***

**Answer**: Thanks for the suggestion. The potential solutions or approaches has been added to lines 391-397: “In the future, we intend to expand our research by studying additional online communities for statin users and collecting demographic information about their users through a survey questionnaire, aiming to generalize our findings. We also intend to explore the applications of large language models to identify the medical concepts from social media texts. In addition, further research is needed to clarify the temporal relationship between statin use and anxiety or depression by integrating this temporal aspect into the EHR validation.”.

***Q7:***

***-sentences are quite long and complex. Consider breaking them down for improved readability
- Ensure consistent capitalization of terms throughout the paper (e.g., "Topic Modeling" vs. "topic modeling")
- Consider adding a brief section on the ethical considerations of using social media data for healthcare research***

**Answer**: We have revised our manuscript to address the above comments. Particularly, the ethical considerations of using social media data for healthcare research was added in the Methods section (lines 117-120).

**Reviewer 2:**

***Q1: The rationale behind using STM is not clear, as STM is used to identify the temporal variation and difference in source data. As far as temporal evidence how did the topics change over time period is not specified.***

**Answer**: Thanks for the comment. We appreciate your observation regarding the typical application of STM for analyzing temporal variation in social media data. Our rationale for employing STM in this study extends beyond its temporal capabilities. We used STM primarily for its ability to incorporate metadata into topic inference, such as user attributes. Therefore, the primary aim of our analysis is to understand the topics related to statin use from clinical perspective, rather than the evolution of topics over time. This is because patients with metabolic syndromes and long-term statin use typically exhibit symptoms and side effects that are relatively stable over time, since metabolic syndrome is a chronic condition characterized by persistent factors such as high blood pressure, insulin resistance, dyslipidemia, and central obesity. We have provided additional rationale for the selection of STM in the Introduction (see response to Reviewer 1, Q1).

***Q2: The Figures provided are not clear, a pipeline/ algorithm describing the entire flow would add value to the manuscript.***

**Answer**:

Thanks for the suggestion. We have added a pipeline overview in Methods section (lines 108-114): “In this study, we began by collecting and preprocessing social media data extracted from Reddit. We then conducted an initial round of STM using a traditional STM pipeline. Next, we enhanced this pipeline by incorporating semantic type information through UMLS concept recognition using MetaMap and performed a second round of STM. Subsequently, we further extended the pipeline by integrating SNOMED precoordinated expressions through concept decomposition, followed by a final round of STM. We then compared the results from the second and final STM rounds. Finally, we validated a newly identified topic from the final round of STM to demonstrate the effectiveness of our approach.”.

***Q3: How do you address the 329 documents which did not have a matching UMLS concepts from the restricted list.***

**Answer**: Thanks for the question. We excluded documents without matching UMLS concepts from the restricted list during the topic modeling process. While we acknowledge that the number of such documents is notable, the majority contained posts where patients primarily discussed their lab test results or sought general advice, with no relevance to our targeted semantic types. This lack of relevance reflects the inherently noisy nature of discussions in this online community, which often include content not directly aligned with the focus of our analysis for understanding the sides effects and concerns of statin users.

***Q4: How is the value of K between 2-6 selected, what is the maximum number of topics generated by the model.***

**Answer**: Thanks for the question. The choice of K (2–6) was based on the nature of the data and experimental evaluations. Discussions among statin users in online communities typically focus on a limited set of symptoms and side effects, so a small number of topics aligns with the content's scope. We tested various K values and found that 2–6 produced coherent and interpretable topics, while larger values led to fragmentation. The model itself does not impose a maximum number of topics; K is defined by the user based on the data and application.

***Q5: Line 124 the authors explain the decomposition of concepts to parent concepts, what is the criteria for identifying the concepts to be decomposed, please explain the logic behind this step.***

**Answer**: We described the criteria for identifying the concepts to be decomposed in the Concept Decomposition Based on SNOMED CT Relationship section: namely, we first identified UMLS concepts with significant word-topic distribution β values (β > 0.02) in each topic and compiled these into a list. Then, we searched each of the UMLS concepts from this list in SNOMED CT and examined whether it had precoordinated expressions. The logic behind the step is that we would like to identify those high-frequency concepts which have parent concepts based on SNOMED relationship and decompose those identified concepts to get a more distinct and meaningful clustering of topics. This ensures semantic clarity while reducing redundancy.

***Q6: In the EHR validation study association between Statin exposure and Mental Health was performed, even though the Subreddit was cholesterol. Why the association study of topic 2 was selected, please validate the reasoning for deciding on the EHR validation study.***

**Answer**: Thanks for the question. We identified three topics: topic 1 (“metabolic syndrome”), topic 2 (“anxiety and depression”), and topic 3 (“muscle pain”). While metabolic syndrome (e.g., hypercholesterolemia) and muscle pain are well-known among statin users, the relationship between statin use and mental health is less explored. To address this gap, we selected topic 2 for validation in the EHR database to assess its relevance and legitimacy.

***Q7: More examples of topics generated and for each topic did the results had metadata text attached to it, please clarify with execution output.***

**Answer**:

Thank you for your comment. Using STM, we generated topics based on the word-topic distribution, which provides the top words associated with each topic. While the model itself does not directly attach metadata text to topics, we can link the topics to metadata by examining the document-topic associations. To address your request, we include examples of the metadata text associated with each topic in the supplementary (S3 text) for further clarity.

**Reviewer 3:**

***Q1: In the Background section, the authors discuss the limitations of traditional topic modeling. However, in the Methods section, they focus on Structural Topic Modeling (STM). It would enhance clarity to explicitly define the gap between STM and existing methods, reinforcing why STM is a better choice for this analysis.***

**Answer**:

Thanks for the comment. We agree that explicitly defining the gap between STM and traditional topic modeling approaches would enhance clarity, and we have revised the descriptions for this in the Introduction section. Unlike other traditional methods, which often struggle with the noisy, dynamic nature of social media data and lack mechanisms to incorporate rich contextual information, STM is specifically designed to address these challenges [1,2,4]. STM allows the integration of document-level metadata, such as user attributes and temporal information, enabling it to effectively uncover latent themes and temporal trends in social media health discussions. Given these strengths, STM is a more robust choice for our analysis, and we consider it well-suited as the baseline approach in our study.

***Q2: As there are several topic modeling approaches available, including STM and LDA, the authors should provide a clear rationale for selecting Structural Topic Modeling (STM) over the more widely used Latent Dirichlet Allocation (LDA). This would make the choice of approach more convincing, particularly in terms of how STM enhances the research in comparison to other methodologies.***

**Answer**:

Thanks for the comment. We have provided additional rationale for the selection of STM (see response to Reviewer 1, Q1). The STM offers advantages over Latent Dirichlet Allocation (LDA) when analyzing social media data, primarily due to its ability to incorporate document-level metadata, such as user attributes. In our study, the document-level metadata includes submission creation time, comment count, score for each submission. This integration enhances the interpretability and relevance of the extracted topics. For instance, the STM allows researchers to estimate topic models that include document-level metadata, providing rich ways to explore topics, estimate uncertainty, and visualize quantities of interest [1,2]. Also, although LDA was popular in many text mining studies, it has been well documented in the empirical literature that LDA is sub-optimal for short texts such as social media and needs to be improved to analyze noisy social media texts [3]. Therefore, we think the traditional STM applied in our study should perform better than the traditional LDA in our dataset. In addition, our focus is to develop a new semantic-type-based topic modeling approach to cluster more meaningful topics in clinical perspective. Hence, the application of other traditional approaches (e.g., LDA) is not necessary and/or may provide less productive results for this dataset.

***Q3: Since this study uses unsupervised approaches, it is important to explain the selection of k (the number of topics) and the initialization process for clustering. The selection of k can greatly influence the results, and more detail here would enhance the paper’s rigor.***

**Answer**: Thanks for the suggestion. Similar to Q4 of Reviewer 2, the choice of K (2–6) was informed by the nature of the data and experimental evaluations. Since discussions among statin users in online communities generally center on a limited set of symptoms and side effects, this suggests that a small number of topics is appropriate for capturing the content's scope. To ensure rigor, we tested a range of K values and found that K=2 to 6 consistently produced coherent and interpretable topics. Larger values led to topic fragmentation, where topics became overly granular and less meaningful. Regarding the initialization process, we used multiple random seeds and assessed the stability of results across runs to mitigate the impact of initialization bias in clustering. This approach ensures that the selected K values lead to robust and replicable topic structures. We believe this methodology balances interpretability with the ability to capture the nuances of the data.

***Q4: In the UMLS Concept Recognition section, the authors mention four semantic types. It would strengthen the paper to explain why these specific types were chosen and how they align with the context of the analysis.***

**Answer**: Thanks for the suggestion. We selected “sosy” (sign or symptom), “dsyn” (disease or syndrome), “mobd” (mental or behavioral dysfunction), and “bpoc” (body part, organ, or organ component) because they are highly relevant to understanding health-related discussions on social media. These semantic types capture key biomedical concepts—such as symptoms, diseases, mental health conditions, and affected body parts—that are central to the health narratives shared in this online community. By focusing on these semantic types, we ensured the extraction of clinically meaningful information while filtering out less relevant terms, improving the precision and relevance of our topic modeling. We have added additional clarification in lines 135-140.

***Q5: In the STM Model Setup section, providing more information on how the number of topics (k) was chosen, and elaborating on the qualitative analysis performed to assess the top words in each topic would improve transparency.***

**Answer**: Thanks for the suggestion. Similar to Q3, the choice of K (2–6) was informed by the nature of the data and experimental evaluations. Since discussions among statin users in online communities generally center on a limited set of symptoms and side effects, this suggests that a small number of topics is appropriate for capturing the content's scope. We tested a range of K values and found that K=2 to 6 consistently produced coherent and interpretable topics. Larger values led to topic fragmentation, where topics became overly granular and less meaningful. We now provide additional detail. Further, we have revised the descriptions in the STM Model Setup section to elaborate on the qualitative analysis (lines 161-166) : “In addition to the quantitative analysis, we conducted a qualitative assessment to evaluate the coherence and interpretability of the topics. For each topic, we examined the top CUIs with the highest probabilities, assessing their semantic and contextual alignment within the topic's overall theme. Furthermore, we reviewed the top 5 original documents associated with each topic to verify whether the extracted CUIs accurately represented the content and context of the documents. This approach ensured that the topics were not only statistically robust but also meaningful and distinct in their interpretations.”.

***Q6: In the Concept Decomposition Based on SNOMED CT Relationships section, it would be beneficial to include an example to demonstrate the concept decomposition process. This will help clarify how granularity issues with SNOMED CT were handled***

**Answer**: Thanks for the suggestion. We have added an example demonstrating the decomposition of “myalgia” to its parent concepts “muscle” and “pain” in this section (lines 173, 177)

***Q7: The process of identifying pre-coordinated expressions is unclear. Explaining whether this was done manually or automatically, and outlining the identification method would enhance understanding. The author cited a JAMIA paper “The use of SNOMED CT, 2013-2020: a literature review.” however, in this paper it said “no evaluation was made as to whether the post-coordinated expression improved the performance of normalization tasks compared to only pre-coordination coding schemes.” it did not indicate the process of pre-coordinate concept identification. Could the authors please explain how you identify pre-coordinated expression?***

**Answer**: Thanks for the question. We appreciate the reviewer highlighting this unsuitable reference, and we have added a new reference “SNOMED CT Starter Guide” to better illustrate the precoordinated expression in SNOMED CT. In practice, we identify the precoordinated expressions from SNOMED CT Browser (<https://browser.ihtsdotools.org/>) and we also added this browser as a reference.

***Q8: The decomposition methodology needs further illustration, especially regarding how it handles pre-coordinated concepts. Without more information, it is difficult to understand how this process works in practice.***

**Answer**: Thanks for the question. We have added more details, as described in our responses to Q6 and Q7. In practice, as described in the Concept Decomposition Based on SNOMED CT Relationships section, the significant UMLS concepts were identified and decomposed using the SNOMED CT hierarchy. For precoordinated concepts, we utilized their parent-child relationships to replace them with parent concepts in the documents (e.g., *myalgia* decomposed into *muscle* and *pain*). This ensures semantic clarity while reducing redundancy. If further details or examples are needed, we are happy to expand the explanation in the revised manuscript.

***Q9: If the method is not intended to be generalized, this should be explicitly addressed in the Discussion section, especially given the complexity of handling a large list of concepts.***

**Answer**: Thank you for the suggestion. We believe our approach could be extended to analyze other conditions or medications on social media. As described in the Concept Decomposition Based on SNOMED CT Relationships section, the method does not require handling a large list of concepts but focuses on a smaller subset with significant word-topic distribution *β* values. Researchers can adjust the *β* threshold to tailor the selection of concepts to their specific needs, making the approach adaptable and scalable depending on the scope of the analysis.

***Q10: Clarifying terms like "word-topic distribution" early in the paper would make the content more accessible to a broader audience.***

**Answer**: Thanks for the suggestion. We have added descriptions for “word-topic distribution” and “topic-document distribution” in the STM Model Setup section (lines 142-146): “STM generates two key probability distributions: the topic-document distribution (θ) and the word-topic distribution (β). The topic-document distribution (θ) represents the probability of each topic within a document, while the word-topic distribution (β) represents the probability of each word within a topic. These distributions are integral to further analysis.”.

***Q11: The comparison of concept decomposition in the STM model is presented without sufficient explanation. If concept decomposition is a key step in improving the topic modeling, this needs to be made more explicit in the manuscript.***

**Answer**: Thanks for the suggestion. We agree that the importance of concept decomposition needs to be described explicitly. Both concept recognition and concept decomposition are key steps in improving topic modeling. Concept recognition ensures that the topic modeling focuses on meaningful semantic types, restricting the analysis to relevant and well-defined biomedical concepts. Concept decomposition, on the other hand, leverages SNOMED’s relationships to replace identified concepts with their parent concepts in the documents. This process enhances semantic clarity by breaking down complex concepts into their fundamental components while reducing redundancy in the data. We highlight the importance of concept decomposition as a key step in improving the topic modeling in the Concept Decomposition Based on SNOMED CT Relationship section and in the third paragraph of the Results section (lines 247-254). We also now more explicitly emphasize the importance of concept decomposition in the Discussion section (lines 342-344). In summary, concept decomposition improves topic modeling by aligning related UMLS concepts within the same topic, reducing overlap and enhancing coherence. This ensures clearer topic boundaries and facilitates the extraction of clinically relevant insights from the data.

**References:**

1.	Roberts M, Stewart B, Tingley D, Airoldi E. The Structural Topic Model and Applied Social Science.

2.	Mishler A, Crabb ES, Paletz S, Hefright B, Golonka E. Using Structural Topic Modeling to Detect Events and Cluster Twitter Users in the Ukrainian Crisis. In: Stephanidis C, editor. HCI International 2015 - Posters’ Extended Abstracts. Cham: Springer International Publishing; 2015. pp. 639–644. doi:10.1007/978-3-319-21380-4_108

3.	Laureate CDP, Buntine W, Linger H. A systematic review of the use of topic models for short text social media analysis. Artif Intell Rev. 2023;56: 14223–14255. doi:10.1007/s10462-023-10471-x

4.	Kang X, Stamolampros P. Unveiling public perceptions at the beginning of lockdown: an application of structural topic modeling and sentiment analysis in the UK and India. BMC Public Health. 2024;24: 2832. doi:10.1186/s12889-024-20160-1
